# CPEB3 Deficiency Elevates TRPV1 Expression in Dorsal Root Ganglia Neurons to Potentiate Thermosensation

**DOI:** 10.1371/journal.pone.0148491

**Published:** 2016-02-25

**Authors:** Sitt Wai Fong, Hsiu-Chen Lin, Meng-Fang Wu, Chih-Cheng Chen, Yi-Shuian Huang

**Affiliations:** Institute of Biomedical Sciences, Academia Sinica, Taipei, Taiwan; Boston Children’s Hospital and Harvard Medical School, UNITED STATES

## Abstract

Cytoplasmic polyadenylation element binding protein 3 (CPEB3) is a sequence-specific RNA-binding protein that downregulates translation of multiple plasticity-related proteins (PRPs) at the glutamatergic synapses. Activity-induced synthesis of PRPs maintains long-lasting synaptic changes that are critical for memory consolidation and chronic pain manifestation. CPEB3-knockout (KO) mice show aberrant hippocampus-related plasticity and memory, so we investigated whether CPEB3 might have a role in nociception-associated plasticity. CPEB3 is widely expressed in the brain and peripheral afferent sensory neurons. CPEB3-KO mice with normal mechanosensation showed hypersensitivity to noxious heat. In the complete Freund's adjuvant (CFA)-induced inflammatory pain model, CPEB3-KO animals showed normal thermal hyperalgesia and transiently enhanced mechanical hyperalgesia. Translation of transient receptor potential vanilloid 1 (TRPV1) RNA was suppressed by CPEB3 in dorsal root ganglia (DRG), whereas CFA-induced inflammation reversed this inhibition. Moreover, CPEB3/TRPV1 double-KO mice behaved like TRPV1-KO mice, with severely impaired thermosensation and thermal hyperalgesia. An enhanced thermal response was recapitulated in non-inflamed but not inflamed conditional-KO mice, with *cpeb3* gene ablated mostly but not completely, in small-diameter nociceptive DRG neurons. CPEB3-regulated translation of TRPV1 RNA may play a role in fine-tuning thermal sensitivity of nociceptors.

## Introduction

Long-term memory (LTM) formation requires *de novo* synthesis of plasticity-related proteins (PRPs) to support long-lasting morphological and functional changes of synapses. Translational control is a seminal means to produce PRPs needed for long-lasting plasticity [1, 2]. The cytoplasmic polyadenylation element binding protein (CPEB) family of RNA-binding proteins and translational regulators contains four members in vertebrates: CPEB1, CPEB2, CPEB3 and CPEB4. Because all are expressed in the brain [3–6], their roles in learning and memory have been investigated. Mice show impaired extinction in spatial and fear LTM with ablation of *cpeb1* [7] but better-consolidated spatial LTM with *cpeb3* knockout (KO) [8]. In contrast, mice with *cpeb4* KO show normal hippocampus-dependent memory [9]. Although the role of CPEB2 in learning and memory has yet to be uncovered, individual CPEB appears to differentially affect learning and memory.

Chronic pain is considered pathological enhancement of neuroplasticity that lowers the threshold for pain signals to be transmitted and sensitizes nociceptive responses. Thus, molecular components affecting PRP synthesis to support cognitive LTM may also contribute to chronic pain development [10]. For example, translation controlled by fragile X mental retardation protein (FMRP) is important for neuronal and intellectual development and for pain responses. FMRP-KO mice show deficits in learning and memory [11] and nociceptive sensitization in peripheral nerve injury-caused hyperalgesia [12]. CPEB1 was expressed almost exclusively in isolectin B4 (IB4)-positive primary afferent nociceptors and was found needed for carrageenan-induced and protein kinase Cɛ-activated hyperalgesic priming and pain memory [13]. Mammalian target of rapamycin (mTOR)-mediated translational control, critical for LTM [14], also affects chronic pain [15, 16]. Most interestingly, a recent study suggested that chronic pain could be rendered labile and mitigated in mice by inhibiting new protein synthesis during the reactivation of spinal pain circuits in a process analogous to memory reconsolidation [17]. Because CPEB3 deficiency results in enhanced plasticity and spatial LTM [8], we investigated whether CPEB3-controlled translation is also central to regulate pain-associated plasticity in this study.

CPEB3 is widely expressed in the central nervous system (CNS) involved in pain sensation, including the somatosensory cortex, thalamus and dorsal horn of the spinal cord. It is also expressed in the peripheral nervous system (PNS), such as small diameter nociceptive neurons (i.e., C-fibers) in dorsal root ganglia (DRG). Because chronic pain could be caused by abnormally enhanced neuroplasticity in PNS, CNS or both, here we examined whether CPEB3-regulated protein synthesis participates in nociceptive responses. CPEB3-KO mice were more sensitive to noxious heat but showed normal thermal hyperalgesia after complete Freund's adjuvant (CFA)-induced inflammation. We then identified the important thermo-sensing and nociceptive molecule TRPV1, whose protein expression was downregulated by CPEB3 in non-inflamed but not inflamed mice. TRPV1, also known as vanilloid receptor 1 (VR1) and belonging to the superfamily of transient receptor potential (TRP) ion channels, is abundantly expressed in nociceptive DRG neurons to mediate pain sensation and transmission [18, 19]. TRPV1 can be activated by multiple physical and chemical stimuli, such as temperature > 43°C, acidic condition and vanilloid compounds (e.g., capsaicin), which results in burning and painful thermosensation. TRPV1 is a polymodal nociceptive sensor whose sensitization and threshold of activation is significantly affected by many inflammatory factors [20]. In addition to post-translational modification to alter the channel activity of TRPV1, TRPV1 was upregulated in lumbar DRG after intraplantar injection of CFA into rat hindpaws [21–23]. Although only the protein but not RNA level of TRPV1 increased after CFA injection [22], the factors that control post-transcriptional TRPV1 synthesis are unclear. Our results demonstrate that CPEB3-confined TRPV1 RNA translation is critical to maintain normal thermal nociception. Inflammation alleviates such a translational repression, contributing to increased TRPV1 synthesis for thermal hyperalgesia.

## Materials and Methods

### Antibodies and Chemicals

Antibodies used were TRPV1 (SC-12498) from Santa Cruz Biotechnology; CGRP (1720–9007) from Biogenesis; NF200 (N0142), and β-actin (A5441), and α-tubulin (T5168) from Sigma-Aldrich. The CPEB3 monoclonal and affinity-purified polyclonal antibodies were described previously [24, 25]. Fura-2 acetoxymethyl ester (Fura 2-AM, F1221), Hoechst 33342 (H3570) and AlexaFluor-conjugated secondary antibodies, including Alexa 488-conjugated donkey anti-rabbit IgG (A21206), Alexa 594-conjugated donkey anti-goat (A11058) and donkey anti-mouse IgG (A21203), were from Invitrogen. DyLight 594-labled GSL (IB_4_, DL-1207) was from Vector Laboratories. CFA, (F5881), capsaicin (M2028) and type Ia collagenase (C9891) were from Sigma-Aldrich.

### Ethics Statement

This study was approved by Institutional Animal Care and Use Committee (IACUC) of Academia Sinica (protocol number: 13-02-512) and compliant with Taiwan Ministry of Science and Technology guidelines for ethical treatment of animals. All of the experimental protocols were performed in accordance with the IACUC guidelines. All efforts were made to minimize the number of animals used and their suffering. C57BL/6 mice were housed under a 12-h light/dark cycle in a climate-controlled room with *ad libitum* access to food and water. CFA and saline were injected into each hindpaw under the plantar surface of mice under isoflurane-inhaled anesthesia. The mice were euthanized with CO_2_ inhalation prior to tissue isolation.

### Animal Genotyping

WT and KO mice were offspring of heterozygous intercrosses. CPEB3-KO and CPEB3/TRPV1 double-KO mice and their controls were littermates from the matings of *cpeb3*^+/-^ x *cpeb3*^+/-^ and *cpeb3*^+/-^*trpv1*^-/-^ x *cpeb3*^+/-^*trpv1*^-/-^, respectively. CPEB3 conditional-WT (cWT) and conditional-KO (cKO) mice were littermates from the matings of *cpeb3*^f/f, Nav-Cre/+^ male mice with *cpeb3*^f/f, +/+^ female mice. TRPV1 heterozygous mice were obtained from the Jackson laboratory (stock no. 003770) and *nav1*.*8*-Cre mice were obtained from Dr. John Wood (University College London). The genotypes were determined by PCR from tail biopsies and by use of the KAPA mouse genotyping kit (KK7302, KAPA Biosystems). Briefly, tail samples were lysed in 20 μl of KAPA extract buffer for 20 min at 75°C, then 5 min at 95–100°C. The DNA sample was then diluted with 60 μl H_2_O, and 0.5 μl of DNA sample was used for a 10-μl PCR reaction. The sense primer, CP3F1 5'-TTTGATCCTTCTGCCTCTCCCTC-3' and two antisense primers, CP3R1 5'-TTGGTACACGACCCTCTTTCCCC-3' and CP3R2 5'-TATGGCTCTGAAGGTCGTGTCCT-3', at a 2:1:1 ratio were used to amplify the CPEB3 WT and KO alleles, respectively. The CP3F1 and CP3R1 primers were also used to distinguish WT and floxed alleles. The two sense primers, TRPVF1 5'-TGGCTCATATTTGCC TTCAG-3' and TRPVF2 5'-TAAAGCGCATGCTCCAGACT-3', and the common antisense primer, TRPVR1 5'- CAGCCCTAGGAGTTGATGGA-3', at a 1:1:2 ratio were used to amplify the TRPV1 WT and KO alleles, respectively. CreF: 5'-TTACCGGTCGATGCAACGAGTGATG-3' and CreR: 5'-GTGAAACAGCATTGCTGTCACTT-3' were used to amplify the *cre* transgene. Each set of primers was then used in multiplex PCR with KAPA2G fast PCR genotyping mix under the following amplification conditions: 95°C for 3 min, 33 cycles of 95°C for 15 s, 55°C for 15 s, 72°C for 15 s and 2 min incubation at 72°C at the end of the run. The amplified products were resolved on a 1% agarose gel.

### Plasmid Construction, *in Vitro* Transcription and Luciferase Reporter Assay

Mouse TRPV1 3'-UTR was PCR amplified from DRG cDNA with the primers 5'-CGGAATTCGGGACACCATGAAGCAGC-3' and 5'-CCGCTCGAGTTGCAGACACACACATAGTTT-3' and cloned into the pcDNA3.1 or pcDNA3.1-FLuc plasmid. The cloning of Arc, PSD95 3'-UTR and myc-CPEB3 was described previously [8, 26]. The RNA used for transfection was synthesized by use of mMessage mMachine T3 and T7 Ultra kits (Invitrogen). Neuro-2a cells were cotransfected with 1.1 μg EGFP, myc-CPEB3 or myc-CPEB3C RNA along with 0.2 μg firefly luciferase RNA appended to the TRPV1 3'-UTR and 0.05 μg *Renilla* luciferase RNA by use of TransMessenger Transfection reagent (Qiagen). At 6 h after transfection, cells were harvested for dual luciferase assay (Promega).

### Assessment of Sensory and Hyperalgesic Responses

Genotypes of 2-month-old male littermates underwent blinded behavior studies to evaluate sensory and hyperalgesic responses. Hot plate test: mice were placed in a Plexiglas cylinder on the surface of a hot plate maintained at 50°C. Thermal sensitivity was determined by measuring the time needed to evoke foot withdrawal responses on the hot plate, including jumping or shaking and lifting hind paws. To avoid tissue injury, the cut-off limit was set at 60 s. von Frey filament test: Mice were placed on an elevated wire mesh platform in a Plexiglass chamber for 1-h acclimation. A series of von Frey filaments with bending forces from 0.008 to 2.0 g were applied in ascending order, beginning with the finest fiber, to the plantar surface of both hind paws. For each paw, a von Frey fiber was applied five times at 30 s intervals. The pain response was scored positive only if the hind paw was removed completely from the platform. The percentage pain response (i.e., paw withdrawal frequency/frequency of abrupt foot lifting) was calculated by dividing the number of paw withdrawals by 5. CFA-induced inflammatory pain model: mice under isoflurane-inhaled anesthesia were injected ipsilaterally with 20 μl CFA (1:1 ratio mixed with saline) or contralaterally with saline (control) into the subcutaneous area of hind paws. A radiant heat source was focused onto the plantar surface of the hind paw. The time that passed before the mouse raised and licked its paw or jumped up was recorded as paw withdrawal latency and used as the index of nociception. A maximal cut-off time of 30 s was used to prevent unnecessary tissue damage. Similarly, mechanical hyperalgesia was measured with von Frey filaments (0.008–0.16 g) as described previously. All data are mean ± SEM. Differences between mouse groups were compared by two-way analysis of variance (ANOVA), except for the hot-plate assay using Student’s *t* test. *P* < 0.05 was considered statistically significant.

### DRG Culture and Calcium Imaging

Lumbar (L) DRG 1–5 isolated bilaterally from a CPEB3 WT or KO mouse were minced briefly in Dulbecco’s modified Eagle’s medium (DMEM), then digested sequentially at 37°C with 0.125% type Ia collagenase (Sigma) for 90 min, then 0.25% trypsin for 20 min. The cell suspension was centrifuged at 1000 rpm for 3 min and the pellet was resuspended in DMEM containing 5% fetal bovine serum (FBS). The ganglia were triturated by use of flame-polished Pasteur pipettes to mechanically dissociate neurons. The dissociated neurons were then plated on collagen-coated coverslips. After cell attachment (about 2–4 h), the medium was replaced with 1:1 ratio of DMEM/Ham’s nutrient mixture F-12 containing 1X penicillin-streptomycin, 10% FBS and 2 mM glutamine. DRG neurons were kept at 37°C in a 5% CO_2_ incubator. Calcium imaging involved the dual-wavelength fluorescent calcium indicator Fura-2AM. The WT and KO DRG neurons at 1 day after plating were washed twice with 1X Hank’s buffered salt solution (HBSS), then incubated in HBSS containing 10μM Fura-2AM and 1% bovine serum albumin (BSA) for 1 h in the incubator. After a 30-min wash with the buffer, the Fura-2AM-loaded neurons were used to record capsaicin-induced calcium influx. Calcium images were acquired under a Zeiss Axiovert 200 inverted microscope at 2-s intervals. Fluorescence ratio at 340 and 380 nm was analyzed by use of MetaFlour software. All procedures were carried out at 37°C, and 1 μM capsaicin was administered by a VC-6 six-channel perfusion system (Warner Instruments).

### RNA Immunoprecipitation (RIP)

DRG isolated from C57BL/6 mice (8–10 weeks old) were snap-frozen in liquid nitrogen and stored at -80°C. The following procedures were carried out at 4°C unless otherwise specified. Frozen DRG collected from 5 mice were homogenized in 0.5 ml RIP buffer containing 50 mM Hepes, pH7.4, 150 mM NaCl, 1 mM MgCl_2_, 0.5% TritonX-100, 1 mM DTT, 10% glycerol, 1X protease inhibitor cocktail (Roche) and 40 U/ml RNase inhibitor. The lysate was rotated for 30 min, then centrifuged at 16,000 *xg* for 5 min to remove insoluble debris. The supernatant was divided and incubated with control or CPEB3 IgG-bound Protein G beads for 3 h with gentle rotation. After washing the beads with 0.5 ml RIP buffer three times, the bound RNA was eluted in the buffer containing 100 mM Tris pH 8.0, 10 mM EDTA, 1% SDS and 200 μg/ml proteinase K and incubated at 55°C for 30 min, followed by phenol/chloroform extraction and ethanol precipitation. The immunoprecipitated RNA was then used for cDNA synthesis and quantitative PCR.

### RNA Isolation, cDNA Synthesis and Quantitative PCR (q-PCR)

Total RNA was extracted by use of TRIzol (Invitrogen) according to the manufacturer's protocol. The cDNA was synthesized with oligo-dT primer and ImPromII Reverse Transcriptase (Promega). Quantitative PCR involved the Universal Probe Library and Lightcycler 480 system (Roche). Data analysis involved the comparative *C*_*t*_ (threshold cycle value) method with non-CPEB3-targeted RNA, GAPDH mRNA as the reference. The PCR primers were for TRPV1, 5'-CCGTGTCACTGGAGAGATCC-3' and 5'-GCCTCTGCAGGAAATACTGG-3'; TRPV2, 5'-CATTCTGCGAGACCTGCT-3' and 5'-TCATTCTGCGAGACCTGCT 3’; TRPA1, 5'-AAACATTGACACATGCTTGGA-3' and 5'-TTTTCCAAGAGGGAAGTGAGG-3'; GAPDH, 5'-GCCAAAAGGGTCATCATCTC-3' and 5'-CACACCCATCACAAACATGG-3'.

### UV-Crosslinking RNA Binding Assay

Recombinant MBP-CPEB3C produced in *E*. *coli* was purified according to the established method [4]. RNA probes for RNA binding assays were labeled by *in vitro* transcription in the presence of α^32^P-UTP. For crosslinking, 20-μl reactions containing 10^6^ cpm labeled RNA, 50 μg heparin, 1 μg recombinant protein and yeast tRNA in 10 mM Hepes, pH 7.4, 50 mM KCl, 1 mM MgCl_2_, 10% glycerol and 0.5 mM DTT were kept on ice or 10 min, then irradiated with 1200 J of UV (254 nm) light for 10 min. UV-crosslinked samples were treated with RNase A and resolved by SDS-PAGE.

### Immunofluorescence Staining

L5 DRG from WT or KO mice were immediately post-fixed in 4% formaldehyde for 60 min at 4°C, then transferred to 30% sucrose overnight at 4°C. DRG were embedded in Tissue-Tek OCT compound and sectioned at 10 μm by use of a Leica cryostat. The slices were stored at -20°C and further fixed in 4% formaldehyde for 10 min right before immunostaining. After three washes with PBS, the slices were blocked for 2 h at room temperature in PBS containing 1% BSA, 5% horse serum and 0.3% Triton X-100, then incubated overnight with the designated primary antibodies at 4°C. Slices were rinsed three times with PBS, then incubated with corresponding AlexaFluor-conjugated secondary antibodies or DyLight 594-conjugated IB_4_ in PBS containing 0.1 mM CaCl_2_, MgCl_2_, and MnCl_2_ at room temperature for 30 min. Tissue sections were washed three times with PBS and mounted with Hoechst 33342 to label nuclei. Images were acquired and quantified under a LSM 700 confocal microscope with a 20X objective lens (Carl Zeiss).

### Immunohistochemistry

Coronal sections of WT and KO male brains after 10-min fixation in 4% formaldehyde and 20-min antigen retrieval in 10 mM sodium citrate buffer, pH 6 at 70°C, were washed twice with Tris-buffered saline (TBS) and permeabilized with 0.2% TritonX-100 in TBS. After three washes with TBS and 1-h blocking in 10% horse serum, the slices were incubated with affinity-purified CPEB3 antibody at 4°C overnight. After washes with TBS, the immunobinding signal was developed by use of the Vectastain Elite ABC kit (Vector labs).

## Results

### CPEB3 Is Expressed in Sensory DRG Neurons and Modulates Thermosensation

**Fig 1:**
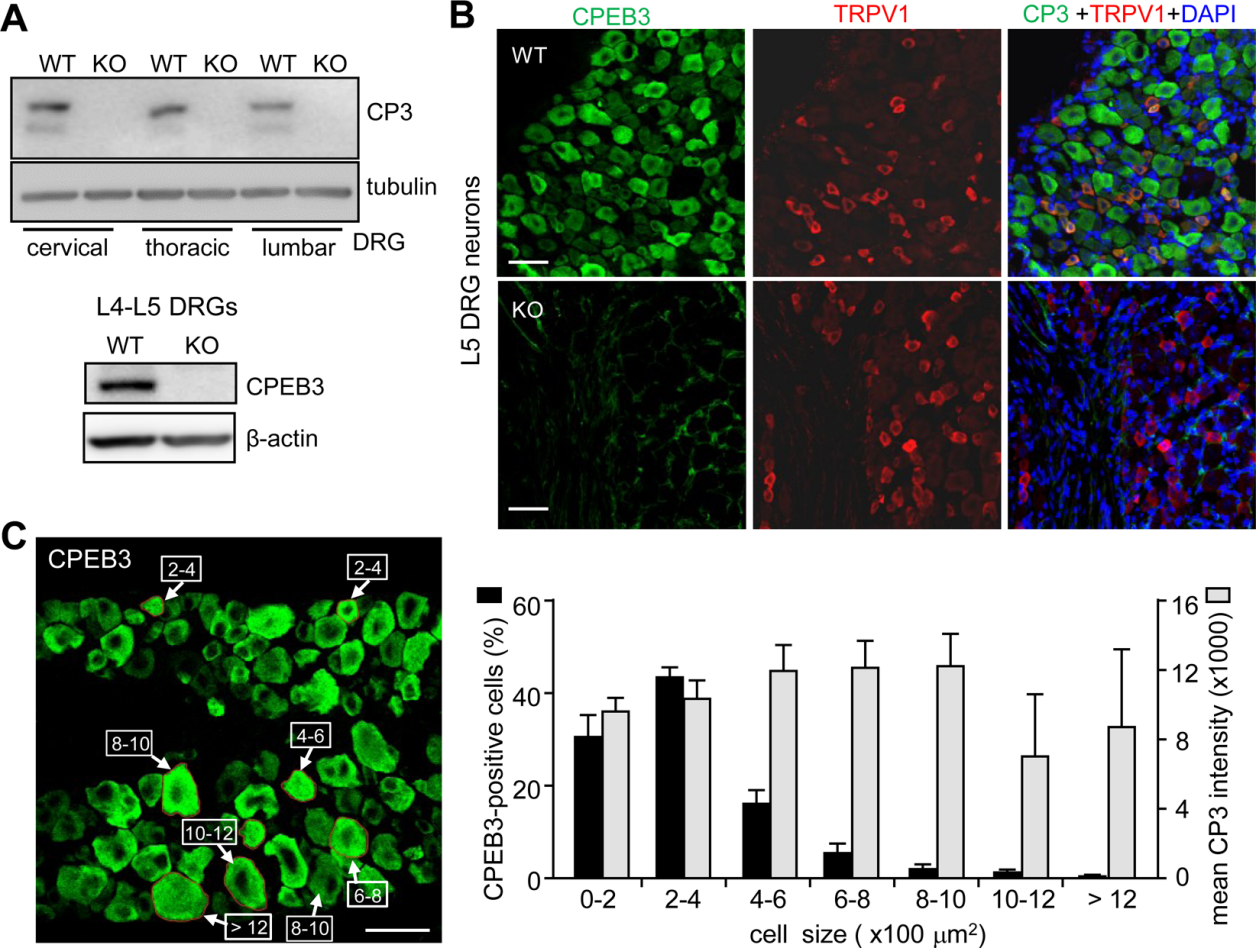
CPEB3 distribution in DRG neurons. (A) CPEB3 (CP3) was detected in cervical, thoracic and lumbar DRG. Lumbar L4-L5 DRGs were isolated and used for western blot or (B) immunostaining assays, both showing CPEB3 immunoreactivity absent in the knockout (KO) DRG neurons. (C) The size of CPEB3-expressing DRG neurons. CPEB3-immunoreactive neurons in several cross-sections of DRG were analyzed for surface area and fluorescence intensity. The percentage of CPEB3-positive cells (black bars) and the mean fluorescence intensity of CPEB3 (grey bars) in DRG neurons of various sizes were expressed as mean ± SEM for L5 DRG isolated from 3 animals. Scale: 50 μm.

In recent human studies, persistent pain is characterized by peripheral and central sensory sensitization as well as network reorganization in brain areas involved in emotion [27]. CPEB3 is broadly expressed in the brain areas (S1A Fig). Immunohistochemistry revealed its distribution in sensory-discriminative areas (e.g., thalamus and somatosensory cortex) and in areas involved in emotion (e.g., anterior cingulate cortex and amygdala). *In situ* hybridization images from the Allen Institute for Brain Science detected broad distribution of CPEB3 RNA in the spinal cord and DRG (S1B Fig). Using CPEB3 wild-type (WT) and KO mice, we confirmed CPEB3 protein expression in mouse spinal cords (S1C Fig) and peripheral DRG (Fig 1A). Primary afferent nociceptive DRG neurons are the first structures to perceive pain stimuli and are involved in the peripheral sensitization that contributes to the development of inflammatory and neuropathic pain. Nevertheless, molecular mechanisms to maintain long-lasting plasticity and sensitization remain largely unexplored.

**Fig 2:**
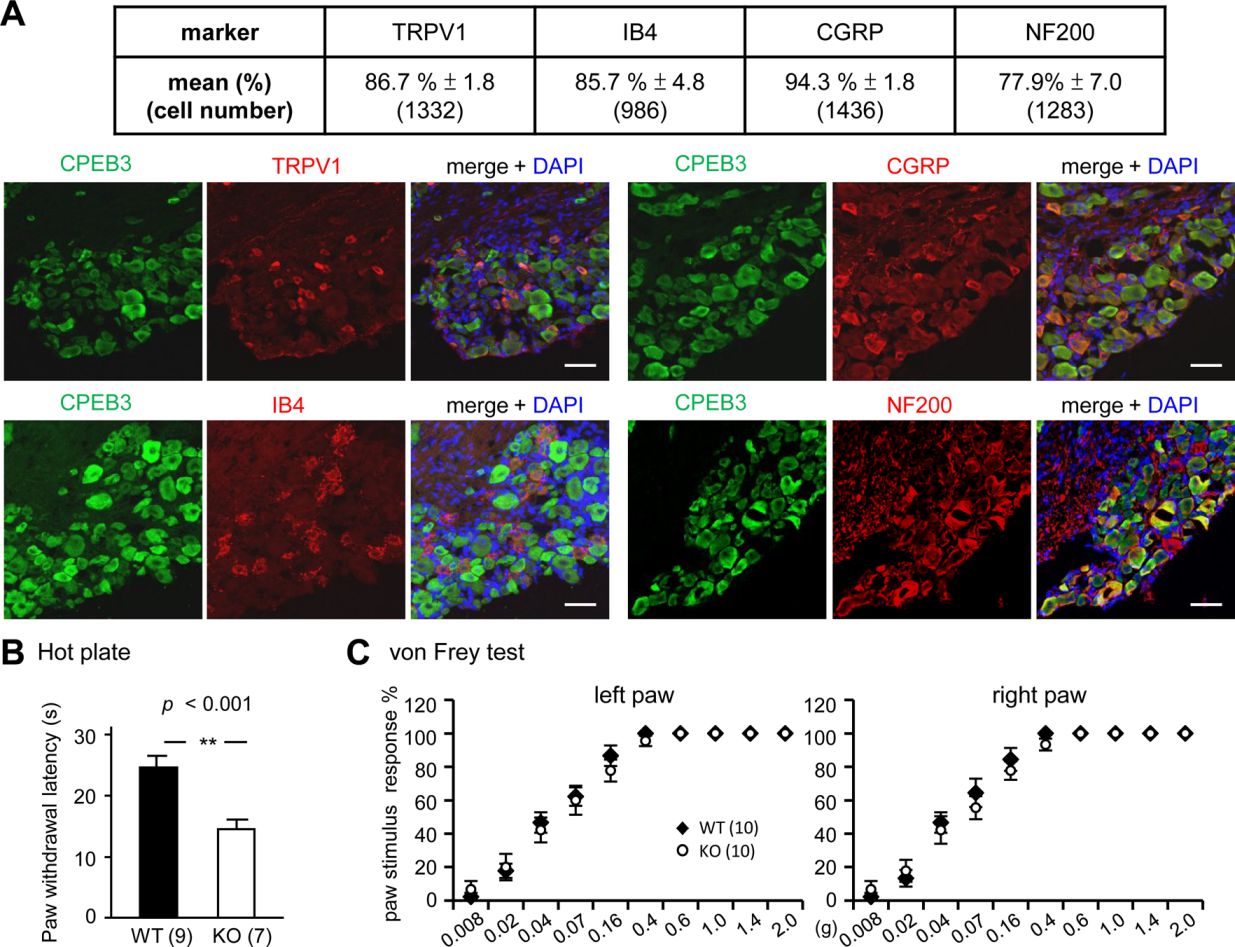
CPEB3-KO mice with normal mechanical sensation were more sensitive to noxious heat than WT littermate mice. (A) Distribution of CPEB3 in subtypes of DRG neurons. DRG isolated from 3 male mice 2 to 3 months old were used for immunostaining of CPEB3 along with one of the denoted marker proteins, TRPV1, calcitonin-gene-related peptide (CGRP), isolectin B4 (IB4) and NF200. The total number of cells analyzed from 3 animals are shown in parentheses. Scale: 50 μm. (B) CPEB3-KO mice showed high thermal sensitivity as indicated by decreased latency to lift paws in the hot plate test (50°C). (C) CPEB3-KO mice displayed normal mechanical sensation in both hindpaws on the von Frey assay. A series of von Frey filaments (0.008–2.0 g) were applied to both hindpaws in ascending order beginning with the finest fiber. For each paw, a von Frey fiber was applied 5 times at 5-s intervals. The pain response was scored positive only if the hindpaw was removed completely from the platform. The percentage of pain response (i.e., paw withdrawal frequency) was calculated by dividing the number of paw withdrawals by 5. The numbers in parentheses represent the number of mice used for assays. Data are expressed as mean ± SEM by Student’s *t* test.

Nociceptive DRG neurons are interesting therapeutic targets to control pain given that they are separated from the CNS by the blood-brain barrier. We first focused on whether CPEB3-controlled translation is central to regulate long-term plasticity and nociceptive response in the PNS. Lumbar (L4-L5) DRG were isolated from WT and KO male littermates and used for western blotting (Fig 1A) and immunostaining (Fig 1B). TRPV1 immunostaining was used to mark some nociceptive neurons (i.e., C-fibers). CPEB3 was widely expressed in DRG neurons, some of which are TRPV1-positive nociceptors. The distribution of the size and fluorescence intensity of CPEB3-positive neurons were analyzed and plotted (Fig 1C). CPEB3 was mostly detected in small-diameter (< 25 μm, surface area < 500 μm^2^) nociceptive DRG neurons, though the mean expression was comparable among neurons of different sizes. Small DRG nociceptive neurons, with mainly unmyelinated axons (i.e., C fibers), can be grouped into peptidergic and non-peptidergic neurons. The former synthesize neuropeptides, such as brain-derived neurotrophic factor, vasoactive intestinal peptide, substance P and calcitonin-gene-related peptide (CGRP), and most express TrkA receptors and respond to nerve growth factor (NGF). The latter express a surface carbohydrate group bound by IB4 and respond to glial-cell line-derived neurotrophic factor (GDNF). Some of both groups of nociceptive neurons express TRPV1 [21, 23, 28]. On immunostaining for CPEB3, along with various DRG neuronal markers, TRPV1, CGRP, IB4 and neurofilament 200 (NF200), to further map its distribution in the subtypes of DRG neurons, we found > 85% of IB4-, CGRP- and TRPV1-positive subpopulations of nociceptors all expressing CPEB3. In addition, CPEB3 was detected in ~78% of NF200-positive DRG neurons (Fig 2A). To examine the thermal and mechanical sensations of CPEB3-KO mice, we used the hot-plate test at 50°C (Fig 2B) and von Frey filament test (Fig 2C), respectively. Notably, the ablation of *cpeb3* gene enhanced thermal nociception (Fig 2B), but not mechanical sensitivity (Fig 2C).

### Moderately Enhanced Inflammatory Hyperalgesia in CPEB3-KO Mice

**Fig 3:**
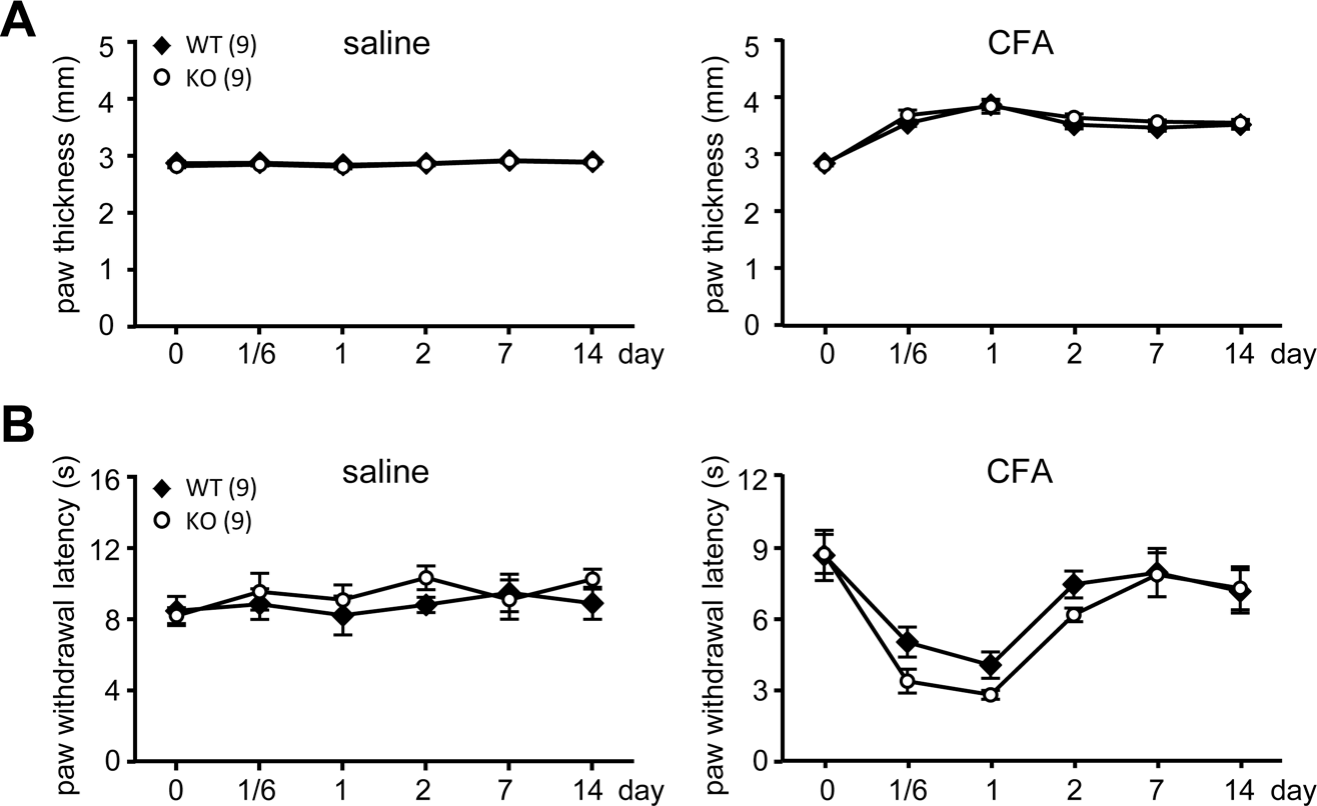
CPEB3-KO mice showed enhanced thermal hyperalgesia. The complete Freund's adjuvant (CFA)-induced inflammatory pain model was used to assess nociceptive responses. CPEB3 WT and KO mice at 2 months old were injected with saline in the left hindpaw and CFA in the right hindpaw to induce inflammatory and swollen responses specifically in the right paw. (A) The thickness of saline- and CFA-injected hindpaws was measured at the indicated time. (B) The mice underwent the radiant heat test to measure the paw withdrawal latency on exposure to radiant heat at the indicated time after the injection of saline and CFA. The number of mice used for the test are in parentheses. Data are mean ± SEM.

**Fig 4:**
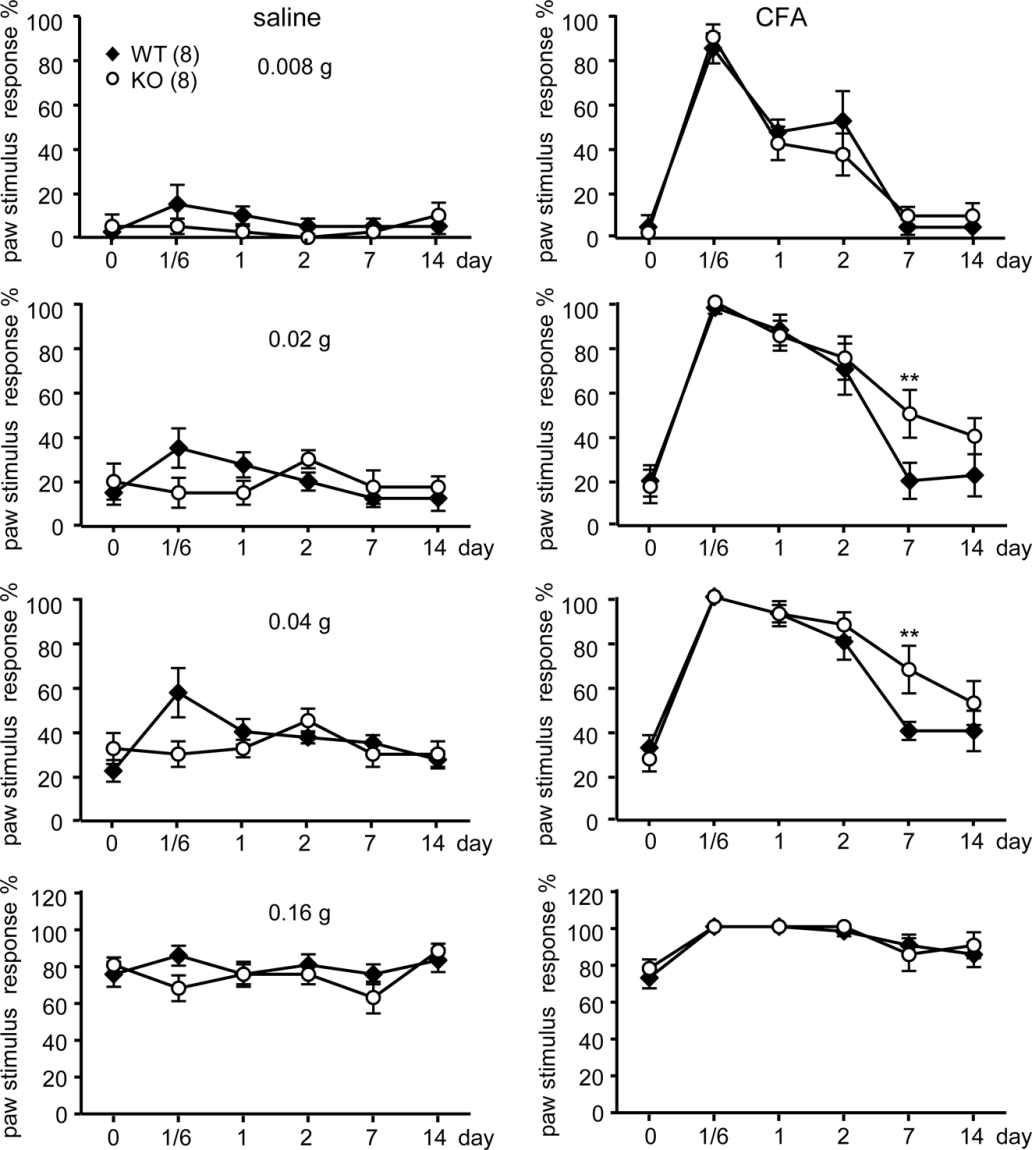
CFA-induced mechanical hyperalgesic responses were comparable between WT and CPEB3-KO mice. CPEB3 WT and KO mice at 2 months of age were injected with saline in the left hindpaw and CFA in the right hindpaw. A series of von Frey filaments (0.008–1.6 g) were applied at both hindpaws in ascending order beginning with the finest fiber. For each paw, a von Frey fiber was applied five times at 5-s intervals. The pain response was scored positive only if the hindpaw was removed completely from the platform. The percentage of pain response (i.e., paw withdrawal frequency) was calculated by dividing the number of paw withdrawals by 5. The numbers in parentheses represent the numbers of mice used for assays. The results were presented as mean ± SEM. * *P <* 0.05 by two-way ANOVA.

The aforementioned results indicate that CPEB3-controlled translation likely modulates gene expression in peripheral nociceptive DRG neurons to regulate sensory responses. In our previous study, CPEB3-KO mice with elevated expression of N-methyl-D-aspartate (NMDA) receptor and PSD95 showed aberrant responses in short- and also long-term plasticity and memory [8]. Thus, we wondered whether abnormal plasticity in the nociceptive neurons would develop in KO mice and affect chronic pain states. We used the CFA-induced inflammation model to investigate the inflammatory pain responses in CPEB3-WT and -KO adult male mice. A single injection of CFA, consisting of heat-inactivated *Mycobacterium tuberculosis* emulsified in mineral oil, into the plantar surface of the hindpaw induced intense and persistent inflammation at the local injection area to cause edema. The thickness of saline-injected left hindpaw and CFA-injected right hindpaw was measured at the indicated times after injection. CFA-caused foot swelling peaked 1 day after injection (Fig 3A) and was associated with the highest thermal hyperalgesic response, determined by paw withdrawal latency on radiant heat exposure (Fig 3B). The KO mice had comparable level of CFA-caused edema as WT mice (Fig 3A) and showed no significant trend of potentiated thermal hyperalgesia from 4 h to 2 days (Fig 3B). By day 7, thermal nociception in both WT and KO mice returned to normal. In the von Frey filament test, neither WT or KO mice showed a potentiated pain response to filaments in saline-injected hindpaw, but both developed mechanical hyperalgesia in the CFA-inflammed hindpaw (Fig 4). Although both WT and KO mice showed similar CFA-evoked mechanical nociception, KO mice exhibited prolonged hyperalgesia when mechanical response in WT mice returned to normal on day 7 (Fig 4).

### Elevated TRPV1 Expression in CPEB3-Depleted DRG

**Fig 5:**
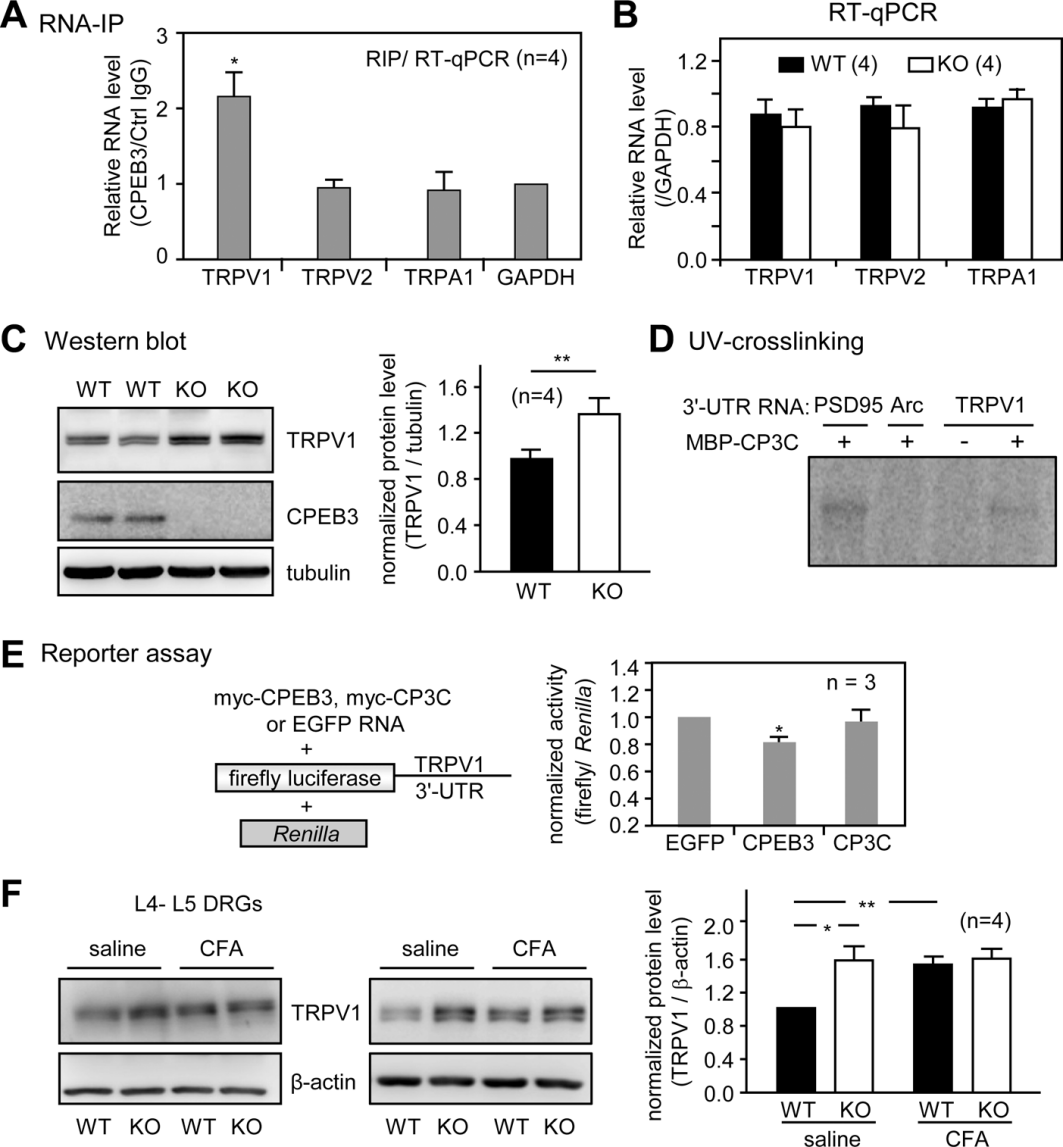
CPEB3 bound to TRPV1 RNA and suppressed its translation. (A) RNA-immunoprecipitation (IP) assay. Total DRG lysates were immunoprecipitated with CPEB3 or control IgG. The pull-down substances were reverse-transcribed for qPCR assay to determine the relative mRNA level of TRPV1, TRPV2, TRPA1 and GAPDH. (B) The relative mRNA levels of TRPV1, TRPV2 and TRPA1 (normalized to that of GAPDH) remained unchanged in CPEB3-KO DRG. (C) Western blot analysis of TRPV1 protein level in the lumbar KO DRG. The quantification from 4 mice per group is shown at the right. (D) Recombinant maltose binding protein (MBP) fused to the C-terminus of CPEB3 RNA-binding domain (MBP-CP3C) was UV-crosslinked with ^32^P-labeled 3'-UTRs of PSD95 (positive control), Arc (negative control) and TRPV1 RNA, RNase treated and then analyzed on SDS-PAGE. (E) RNA reporter assay. Neuro-2a cells were transfected with RNAs encoding myc-CPEB3, myc-CPEB3C or EGFP, combined with the firefly luciferase appended to the TRPV1 3'-UTR and *Renilla* luciferase. Normalized luciferase activity (firefly/*Renilla*) was calculated and expressed as mean ± SEM from 3 independent experiments. (F) CFA injection dampened CPEB3-inhibited TRPV1 expression. The hindpaws of 4 pairs of WT and KO male mice were injected with saline or CFA. L4-L5 DRG were isolated 1 week later for immunoblotting of TRPV1 and β-actin. Data are mean ± SEM. *,** *P <* 0.05 and *P <* 0.01, respectively, by Student’s *t* test.

The behavior studies suggest that CPEB3-controlled translation may modulate nociception, particularly thermally evoked pain-related behaviors. We wondered about the molecular changes in CPEB3-depleted DRG neurons that could lead to elevated thermal response to noxious heat (Fig 2B). There are at least six temperature sensors expressed in sensory neurons and gated at specific temperatures, including TRPV1 (> 43°C), TRPV2 (> 52 ^o^C), TRPV3 (> 30–39 ^o^C), TRPV4 (> 25–35°C), TRPM8 (< 20–28 ^o^C) and TRPA1 (< 17 ^o^C) [29–35]. Compared with TRPV3 and TRPV4, TRPV1 and TRPV2 are more abundantly expressed in DRG and respond to noxious heat [35, 36]. Thus, we first used RNA-immunoprecipitation to examine the association between CPEB3 and transcripts encoding heat-sensing channels, TRPV1 and TRPV2, and a cold-sensing channel, TRPA1. Only TRPV1 but not TRPV2, TRPA1 or the non-target control glyceraldehyde 3-phosphate dehydrogenase (GAPDH) RNA was co-precipitated with CPEB3 (Fig 5A). TRPV1 is a critical thermo-sensing mediator and expressed in many small-sized nociceptive DRG neurons. TRPV1-KO mice showed impaired acute heat nociception in the hot plate test and CFA-induced thermal but not mechanical hyperalgesia [19, 23, 37]. In contrast, TRPV2 is expressed in a subpopulation of medium- to large-diameter myelinated sensory neurons [29], whose depletion in mice has no effect on thermal and mechanical nociception [38]. The high degree of co-expression of CPEB3 and TRPV1 in the subpopulation of DRG neurons (Fig 2A) and enhanced thermo-nociception (Fig 2B) in KO mice also suggest the possible role of CPEB3 in suppressing translation of TRPV1 RNA. The mRNA levels of TRPV1, TRPV2, and TRPA1 remained unchanged between WT and KO (Fig 5B), but TRPV1 protein level significantly increased in KO lumbar DRG (Fig 5C) and in lumbar sciatic nerves (S2A Fig) and spinal cord (S2B Fig). Moreover, CPEB3 directly bound to the TRPV1 3'-untranslated region (UTR) and repressed translation of a reporter RNA appended to this 3'-UTR sequence. The radiolabeled 3'-UTRs of TRPV1, PSD95 (a positive control) and Arc (a negative control) mRNA were subjected to *in vitro* UV-crosslinking with the C-terminal RNA-binding domain of CPEB3 fused to maltose-binding protein (MBP-CP3C). The *in vitro* binding and RNA reporter assays revealed that CPEB3 directly bound to the 3'-UTR of TRPV1 RNA (Fig 5D) and suppressed about 20% of the translation of reporter RNA in Neuro-2a cells (Fig 5E). TRPV1 protein but not RNA level increased about 1.5- to 2-fold in lumbar DRG from 1 to 7 days after intraplantar injection of CFA to rat hindpaw [22]. Because the difference in thermo-nociception between WT and KO groups is more evident in the basal (Fig 2B) than inflammatory state (Fig 3B), we wondered whether the difference in TRPV1 expression was less obvious between the two groups after CFA-induced inflammation. L4-L5 DRG were isolated from WT and KO mice 7 days after saline or CFA injection in the ipsilateral side. Indeed, CFA injection increased TRPV1 protein level in WT but not KO DRG (Fig 5F).

### Elevated TRPV1 Expression in DRG Neurons Accounts for Thermal Hypersensitivity in CPEB3-KO Mice

**Fig 6:**
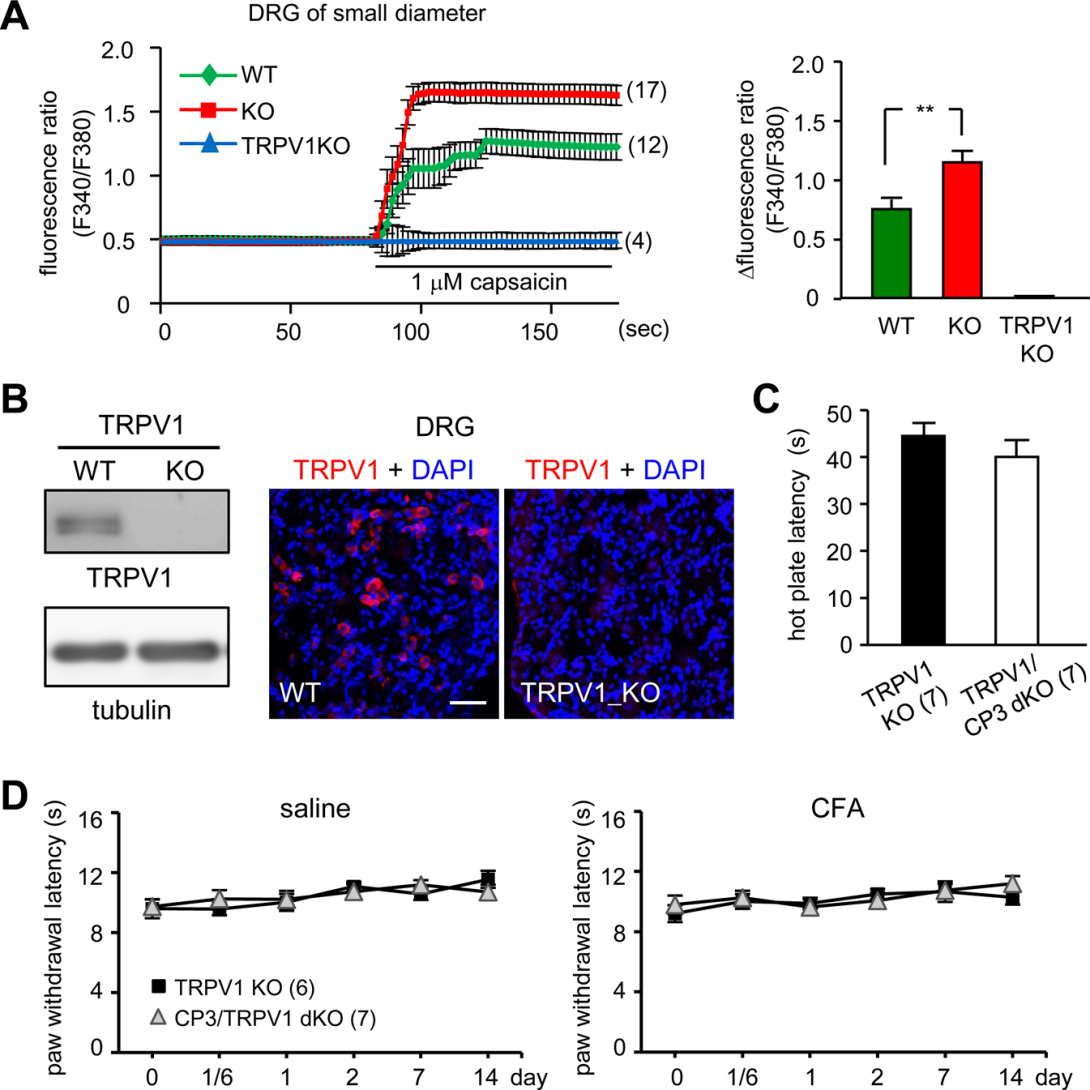
Increased TRPV1-mediated calcium influx in CPEB3-KO DRG neurons. (A) Fura2-AM-loaded DRG neurons were subjected to calcium imaging and treated with capsaicin as indicated. The change in [Ca^2+^] influx was monitored by the fluorescence ratio at 340/380 nm (F/F_0_) and plotted against time. Only small-sized nociceptive neurons were analyzed. Data are mean ± SEM from 4 independent cultures. ** *P <* 0.01 by Student’s *t* test. TRPV1KO DRG neurons was a control to confirm that capsaicin-activated calcium influx is mediated via TRPV1. (B) Immunoblots of DRG lysates and immunostaining of DRG sections prepared from WT and TRPV1-KO mice confirmed the specificity of TRPV1 antibody. In the *trpv1*^-/-^ genetic background, CPEB3 deficiency (i.e., CP3/TRPV1 double KO) did not potentiate (C) thermal sensitivity in the hot plate assay and (D) CFA-induced thermal hyperalgesia. Data are mean ± SEM.

Elevated TRPV1 protein expression in KO DRG neurons was also reflected by an increase in capsaicin-activated TRPV1-mediated calcium influx (Fig 6A). Fura2-AM-filled WT and KO DRG neurons were used to monitor calcium influx [Ca^2+^]_i_ via opening of the TRPV1 channel on ratio-matrix analysis (F/F_0_). Capsaicin-activated [Ca^2+^]_i_ in small-diameter DRG neurons was greater in CPEB3-KO than in WT and completely absent in TRPV1 KO neurons (Fig 6A). Using the TRPV1-KO mice, we also confirmed that the antibody indeed detected TRPV1 in DRG neurons in both immunoblotting and immunostaining assays (Fig 6B). The *cpeb3* and *trpv1* genes are located in two different chromosomes (19 vs. 11, respectively), so we produced *cpeb3*^*+/+*^ *trpv1*^*-/-*^ (i.e., TRPV1 KO) and *cpeb3*^*-/-*^*trpv1*^*-/-*^ (i.e., CPEB3/TRPV1 double KO) mice and found comparable and severely defective thermo-nociception (Fig 6C and 6D). Thus, TRPV1 is the key target affected by CPEB3 to enhance thermal nociceptive responses in CPEB3-KO mice.

**Fig 7:**
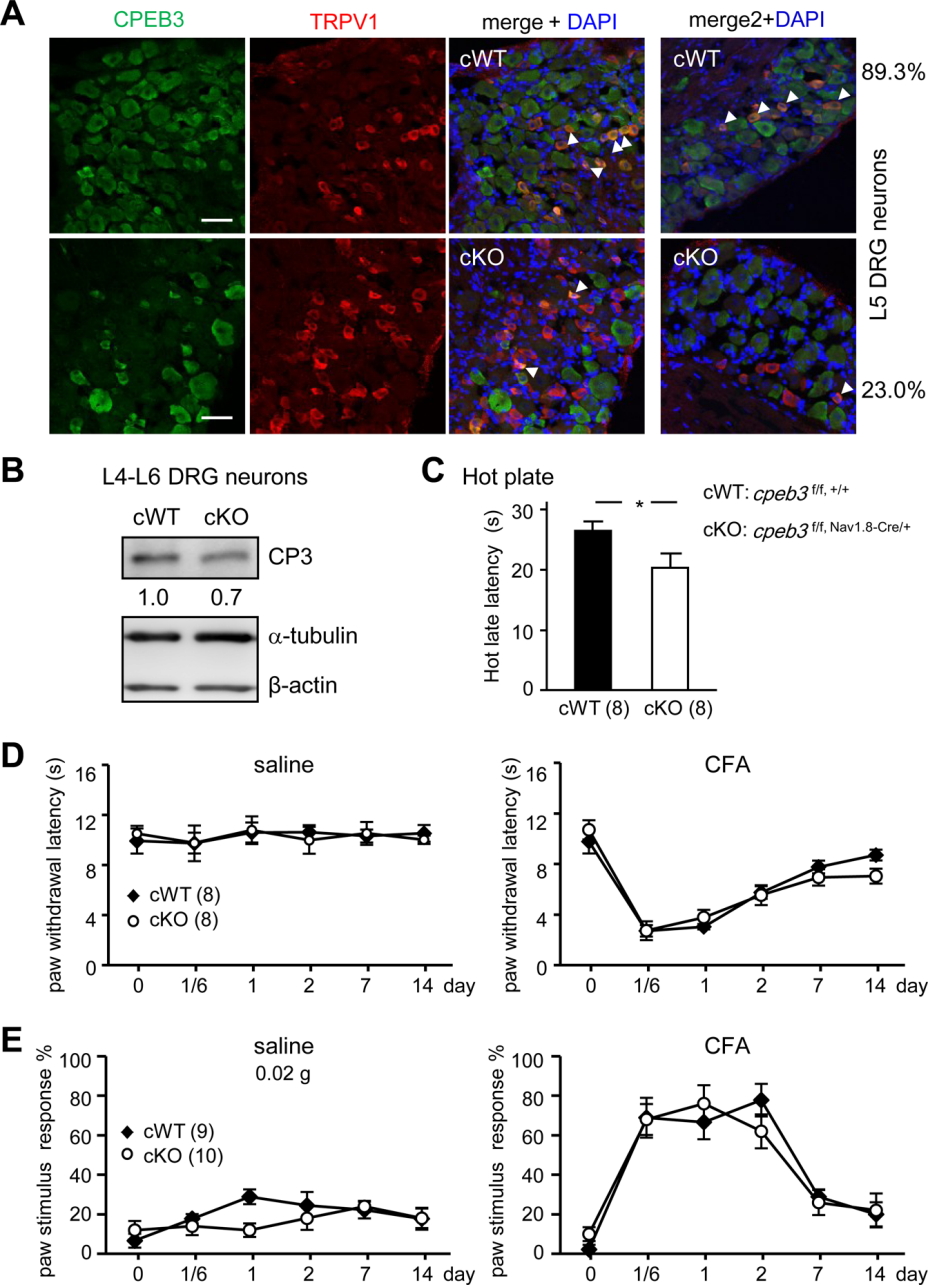
Specific depletion of CPEB3 in nociceptive DRG neurons affected thermal sensitivity. The *cpeb3* gene was ablated in nociceptive DRG neurons by crossing floxed CPEB3 (*cpeb3*^f/f^) mice with the sodium channel (Nav)1.8-Cre mouse line to generate the conditional WT and KO mice (cWT: CPEB3^f/f, +/+^ and cKO: CPEB3^f/f, Nav1.8-Cre/+^). (A) Immunostaining data indicated that cKO L5 DRG had only 23% TRPV1-positive neurons expressing CPEB3 as compared with 89% in cWT L5 DRG. Scale: 50 μm. (B) Western blot assay showing ~ 30% reduction in the CPEB3 level in cKO DRG. CPEB3 cKO mice showed (C) potentiated thermal sensitivity in the hot plate assay but normal CFA-induced (D) thermal and (E) mechanical hyperalgesic responses.

To further determine whether elevated TRPV1 expression in nociceptive DRG neurons is sufficient to affect thermal nociception and CFA-evoked thermal hyperalgesia, mice carrying *cpeb3* floxed alleles (*cpeb3*^f/f^) were crossed with the *nav1*.*8*-*cre* mouse line to generate CPEB3 conditional-KO (cKO, *cpeb3*^f/f, cre/+^) mice. Previous studies indicated that the Nav1.8 sodium channel is specifically expressed in small-diameter DRG neurons [39]. Under this conditional ablation, the proportion of CPEB3-detectable cells in TRPV1-expressing neurons decreased from 89% to 23% (Fig 7A, averaged data from two animals per group). About 70% of CPEB3 protein remained in cKO DRG (Fig 7B), which again shows that CPEB3 is not just restricted to nociceptive small-sized cells (Figs 1C and 2A). Indeed, CPEB3 was detected in NF200-positive DRGs of those cKO mice (S3 Fig). Although the cKO mice still displayed thermal hypersensitivity (Fig 7C), CFA-induced thermal (Fig 7D) and mechanical (Fig 7E) hyperalgesic responses were similar to those in their conditional-WT littermates (cWT, *cpeb3*^f/f, +/+^). Our data demonstrate that CPEB3 functions as a negative regulator in thermal responses by confining TRPV1 RNA translation. Disruption of this inhibitory mechanism restrictedly in a subset of small-diameter DRG neurons is sufficient to affect thermal sensitivity to noxious heat.

## Discussion

In this study, we characterized the expression pattern of CPEB3 in DRG neurons in mice. Almost all DRG neurons, including those of small-diameter nociceptive neurons, express CPEB3. The high degree of co-expression of CPEB3 and TRPV1 in the subpopulation of DRG neurons implicates a role of CPEB3 in suppressing TRPV1 RNA translation. CPEB3 deficiency causes elevated TRPV1 expression in DRG and the spinal cord, which accounts for increased thermosensation. Although nociceptor-specific depletion of CPEB3 is sufficient to cause hypersensitivity to noxious heat, the difference between cWT and cKO mice (Fig 7C) is much less than that between WT and KO mice (Fig 2B), which suggests that CPEB3-suppressed TRPV1 expression in the spinal cord or the remaining 23% of TRPV1^+^ DRG neurons also contributes to thermal nociception.

TRPV1 is predominantly expressed in small- and medium-sized nociceptive sensory neurons [30, 40] and its activity can be enhanced by pro-inflammatory molecules such as prostaglandins, bradykinin, adenosine triphosphate and nerve growth factor (NGF) [18, 20, 41, 42]. Protein kinase A (PKA) and protein kinase C (PKC) in response to those inflammatory stimuli phosphorylate TRPV1 to enhance its channel function [42–45]. In addition, TRPV1 expression increases upon inflammation induced by various agents [21–23, 46–49]. Following CFA-evoked inflammation, differentially increased NGF and GDNF at different time windows promote TRPV1 synthesis in TrkA-expressing peptidergic and IB4^+^-nonpeptidergic nociceptive neurons, respectively, to cause thermal hyperalgesia [46]. Although NGF-facilitated TRPV1 expression depends on p38 kinase signaling and is regulated at the protein level [22], no translational regulator has been identified to control TRPV1 synthesis.

Our results indicate that TRPV1 RNA translation is negatively regulated by CPEB3 to modulate thermal sensitivity. In response to CFA-induced inflammation, CPEB3 no longer represses TRPV1 synthesis (Fig 5F). Such an inflammation-induced translational derepression mechanism also supports the thermal nociception results, which show greater disparity between CPEB3 WT and KO groups in the basal (Fig 2B) than inflammatory state (Fig 3B). CPEB3 is cleaved by calpain 2 in NMDA-stimulated cultured cortical/hippocampal neurons and the degradation of CPEB3 is accompanied by the elevated expression of CPEB3's target, epidermal growth factor receptor [25]. However, CPEB3 level in CFA-injected ipsilateral DRGs was not downregulated (S4 Fig). A recent study indicated that inflammatory sensitization of DRG nociceptors requires activation of NMDA receptors in DRG satellite cells but not neurons [50]. Calcium imaging results also confirmed that NMDA induced a transient calcium influx in cultured DRG satellite cells but not neurons. Thus, inflammation might induce posttranslational modification of CPEB3 to ameliorate its repressor function, instead of by NMDA signaling-induced CPEB3 degradation. How inflammation causes derepression of TRPV1 RNA translation and whether NGF and GDNF signaling pathways act upon CPEB3 to alleviate its repressor function remain to be investigated. Because CFA-induced mechanical hyperalgesia is normal in TRPV1-KO mice [19], the molecular changes responsible for moderately prolonged mechanical hyperalgesia in CPEB3-KO mice need to be further characterized. CPEB3 is also expressed in large DRG neurons, including those involved in the sensations of touch and proprioception. Nevertheless, CPEB3 KO mice respond to touch and have normal motor coordination and swimming activity in the rotarod and Morris water maze tasks, respectively [8]. Capsaicin-induced calcium influx in large DRG neurons is extremely low as expected, so we did not compare the difference between WT and KO cultures. Interestingly, recent studies reported that the expression and function of TRPV1 increase in large DRG neurons in the streptozotocin-induced diabetic rats, a model for diabetic sensory neuropathy [51]. Moreover, capsaicin treatment-induced oxidative stress and cell injury in large DRG neurons depend on caspase and calpain [52]. Whether CPEB3 controls TRPV1 RNA translation in large DRG neurons in diabetic or other neuropathic pain models remains to be tested.

Recent studies reported that TRP channels might be involved in psychiatric disorders such as anxiety and depression. Both TRPV1- and TRPA1-KO mice showed reduced anxiety-related behaviors [53, 54]. Interestingly, CPEB3-KO mice exhibited anxiety-like responses with reduced exploratory behaviors in the open field assay [8], which suggests that CPEB3-suppressed TRPV1 expression may also occur in the brain. Using the same antibody that could specifically detect TRPV1 in DRG and the spinal cord, we found an immunoreactive band of the right size in the hippocampus and cortex. The signal intensity of this band remained unchanged in the absence of CPEB3 (S5A Fig). Nevertheless, this immunoreactive signal is not TRPV1 because it was also detected in the TRPV1-KO cortex (S5B Fig). Thus, whether CPEB3-regulated TRPV1 RNA translation exists in the brain to affect anxiety or other cognitive function remains to be determined.

Treatment of pain is an unmet medical need. The increased sensitivity of peripheral nociceptive neurons is a major cause of amplified sensation of pain following injury. This plasticity is thought to contribute to the maintenance of chronic pain states and requires changes in gene expression to support functional enhancement in nociceptive neurons after injury. Studies of genetic deletion and pharmacological blockade of TRPV1 [19, 20, 49] demonstrate that TRPV1 is essential for thermal nociception or inflammation-induced hyperalgesia. Thus, TRPV1 is a promising target for relieving inflammatory pain. Nevertheless, many developed drugs fail to satisfy analgesic effects or have severe off-target or side effects [41, 55, 56]. For example, drugs acting at TRPV1 can ameliorate neuropathic pain [41, 55] but can also induce unwanted hyperthermia [57]. Thus, understanding the etiology of pain and associated plasticity may identify new opportunities for treating pain. If we can elucidate how inflammation alleviates CPEB3-repressed TRPV1 expression, intervening in this de-repression should dampen TRPV1 synthesis and peripheral sensitization without directly blocking the TRPV1-sensing function to jeopardize the defensive response to noxious stimuli. The findings here provide a novel mechanism to modulate TRPV1 signaling via translational control.

## Supporting Information

S1 Fig CPEB3 distribution in the sensory nociceptive system. (A) Immunohistochemistry of coronal brain slices with affinity-purified polyclonal CPEB3 antibody, used previously to detect no immunostained signal in knockout (KO) tissue. Brain areas: 1, anterior cingulate cortex; 2, somatosensory cortex; 3, thalamus; 4, amygdala. Scale, 1 mm. (B) The in situ hybridization images were from the Allen Institute for Brain Science and show CPEB3 RNA in dorsal and ventral horns of spinal cord and dorsal root ganglia (DRG). (C) Western blot analysis with wild-type (WT) and KO lumbar spinal cord confirmed the expression of CPEB3 in the spinal cord.
(TIF)

S2 Fig Increased TRPV1 level in lumbar sciatic nerves and spinal cords. (A) Lumbar sciatic nerves and (B) lumbar spinal cord were isolated from CPEB3 WT and KO male mice for western blot analysis of TRPV1 and β-actin. Data are mean ± SEM from 3–4 animals per group. *P < 0.05 by Student’s *t* test.
(TIF)

S3 Fig CPEB3 remained in large DRGs of cKO mice. L4-L5 DRGs isolated from conditional WT and KO mice (cWT: CPEB3^f/f, +/+^ and cKO: CPEB3^f/f, Nav1.8-Cre/+^) were used for nuclear staining of DAPI and immunostaining of CPEB3, TRPV1 and NF200. TRPV1-immunostained signal was pseudo-colored in magenta. Scale: 50 μm.
(TIF)

S4 Fig CPEB3 level in lumbar DRG with or without intraplantar injection of CFA. The hindpaws of WT and KO male mice were injected with saline or CFA. L4-L5 DRG were isolated a week later for immunoblotting of CPEB3 and β-actin.
(TIF)

S5 Fig TRPV1-immunoreactive signal in the brain is non-specific. (A) Hippocampal and cortical tissues isolated from CPEB3 WT and KO male mice were used for immunoblotting with TRPV1, tubulin and β-actin antibodies. TRPV1 signals in WT and KO tissues were comparable. (B) Although TRPV1-immunoreactive signals in cortex and DRG were of the same molecular weight, the signal in the cortex was non-specific and still present in the TRPV1 KO cortex.
(TIF)
