# Supplementary figures and images for: CPEB3 Deficiency Elevates TRPV1 Expression in Dorsal Root Ganglia Neurons to Potentiate Thermosensation
Source: PLoS One. 2016 Feb 25;11(2):e0148491. doi: 10.1371/journal.pone.0148491 (PMC4767414; doi:10.1371/journal.pone.0148491)

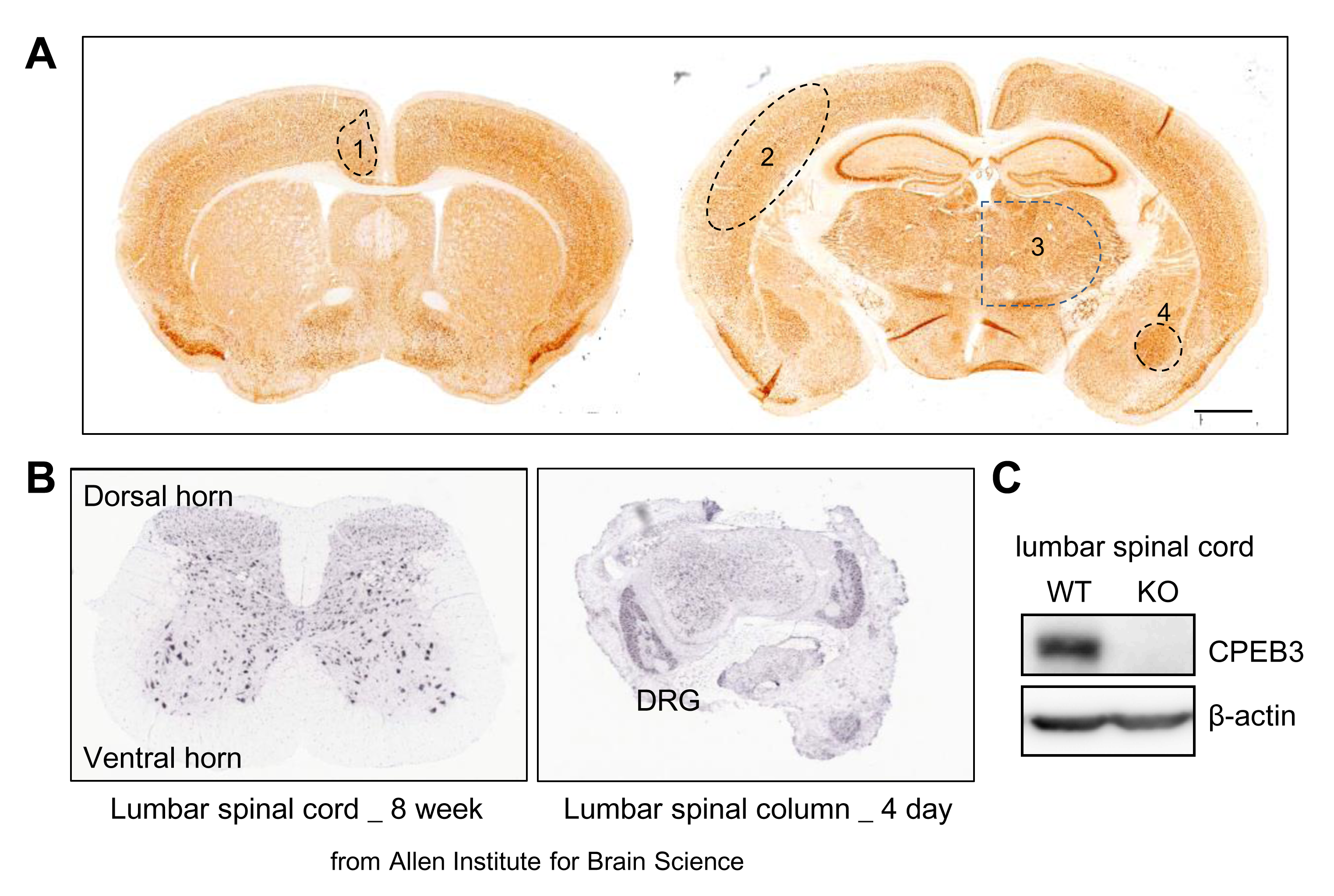

Supplement: S1 Fig — (A) Immunohistochemistry of coronal brain slices with affinity-purified polyclonal CPEB3 antibody, used previously to detect no immunostained signal in knockout (KO) tissue. Brain areas: 1, anterior cingulate cortex; 2, somatosensory cortex; 3, thalamus; 4, amygdala. Scale, 1 mm. (B) The in situ hybridization images were from the Allen Institute for Brain Science and show CPEB3 RNA in dorsal and ventral horns of spinal cord and dorsal root ganglia (DRG). (C) Western blot analysis with wild-type (WT) and KO lumbar spinal cord confirmed the expression of CPEB3 in the spinal cord. (TIF) [file pone.0148491.s001.tif]

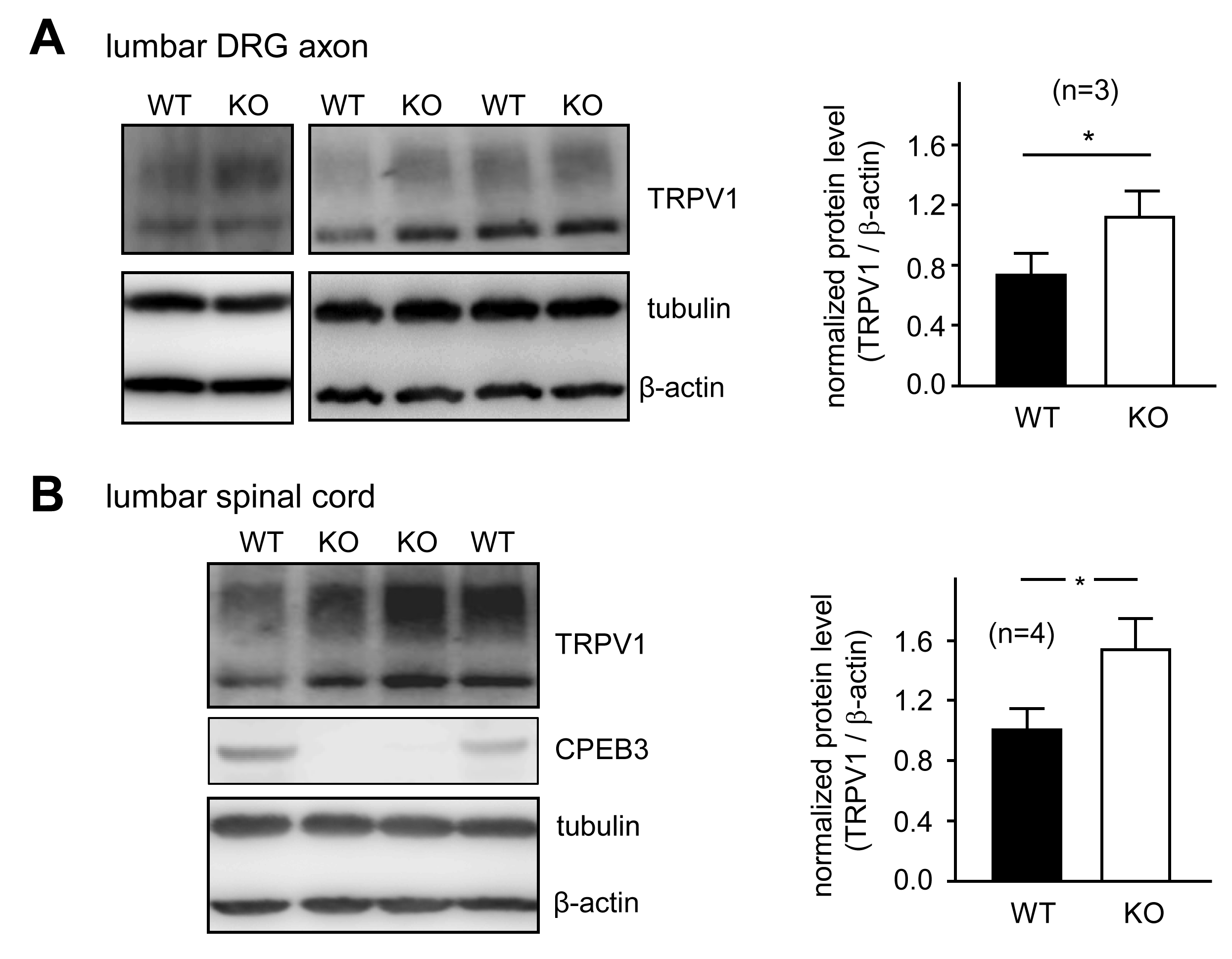

Supplement: S2 Fig — (A) Lumbar sciatic nerves and (B) lumbar spinal cord were isolated from CPEB3 WT and KO male mice for western blot analysis of TRPV1 and β-actin. Data are mean ± SEM from 3–4 animals per group. *P < 0.05 by Student’s t test. (TIF) [file pone.0148491.s002.tif]

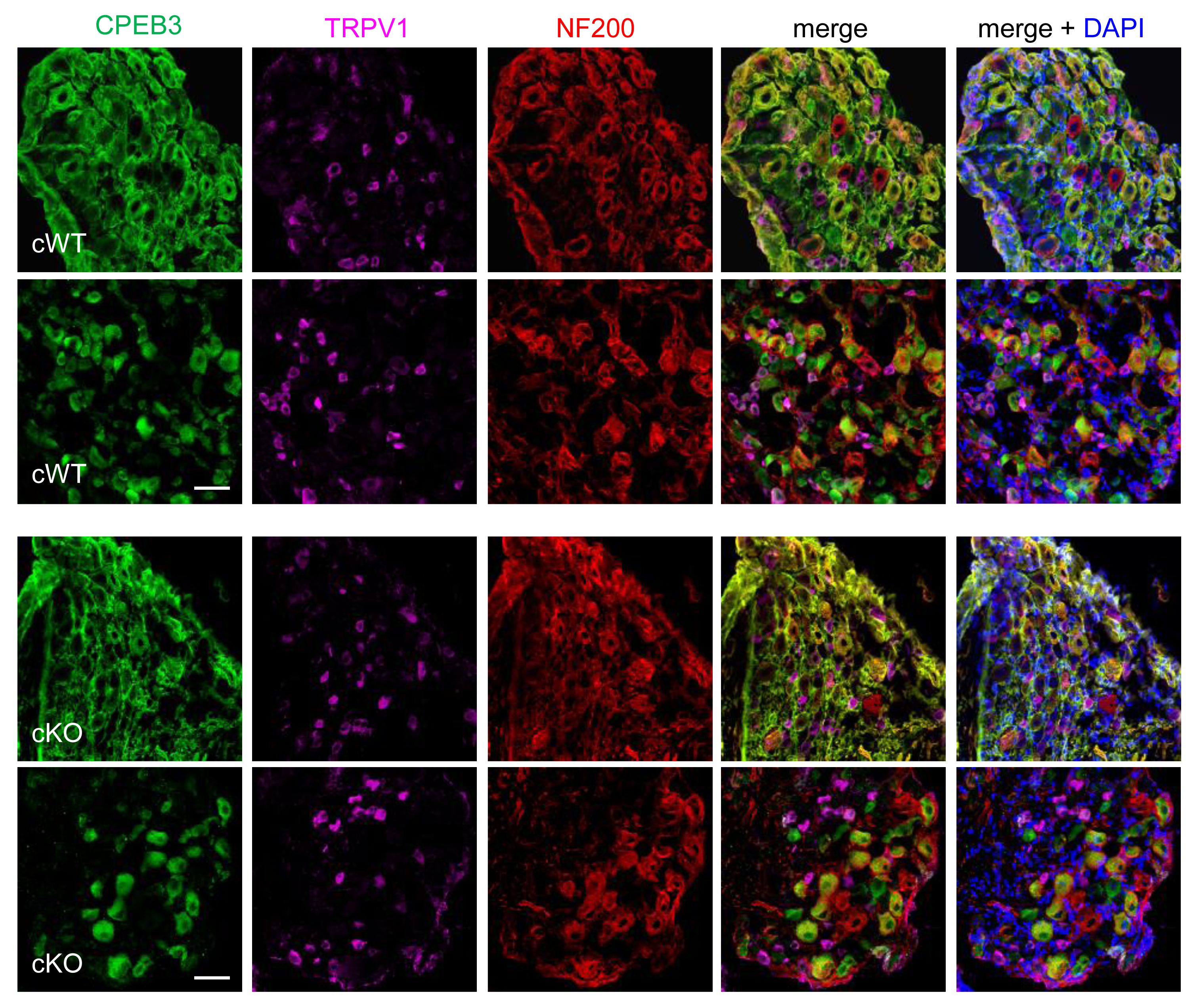

Supplement: S3 Fig — L4-L5 DRGs isolated from conditional WT and KO mice (cWT: CPEB3f/f, +/+ and cKO: CPEB3f/f, Nav1.8-Cre/+) were used for nuclear staining of DAPI and immunostaining of CPEB3, TRPV1 and NF200. TRPV1-immunostained signal was pseudo-colored in magenta. Scale: 50 μm. (TIF) [file pone.0148491.s003.tif]

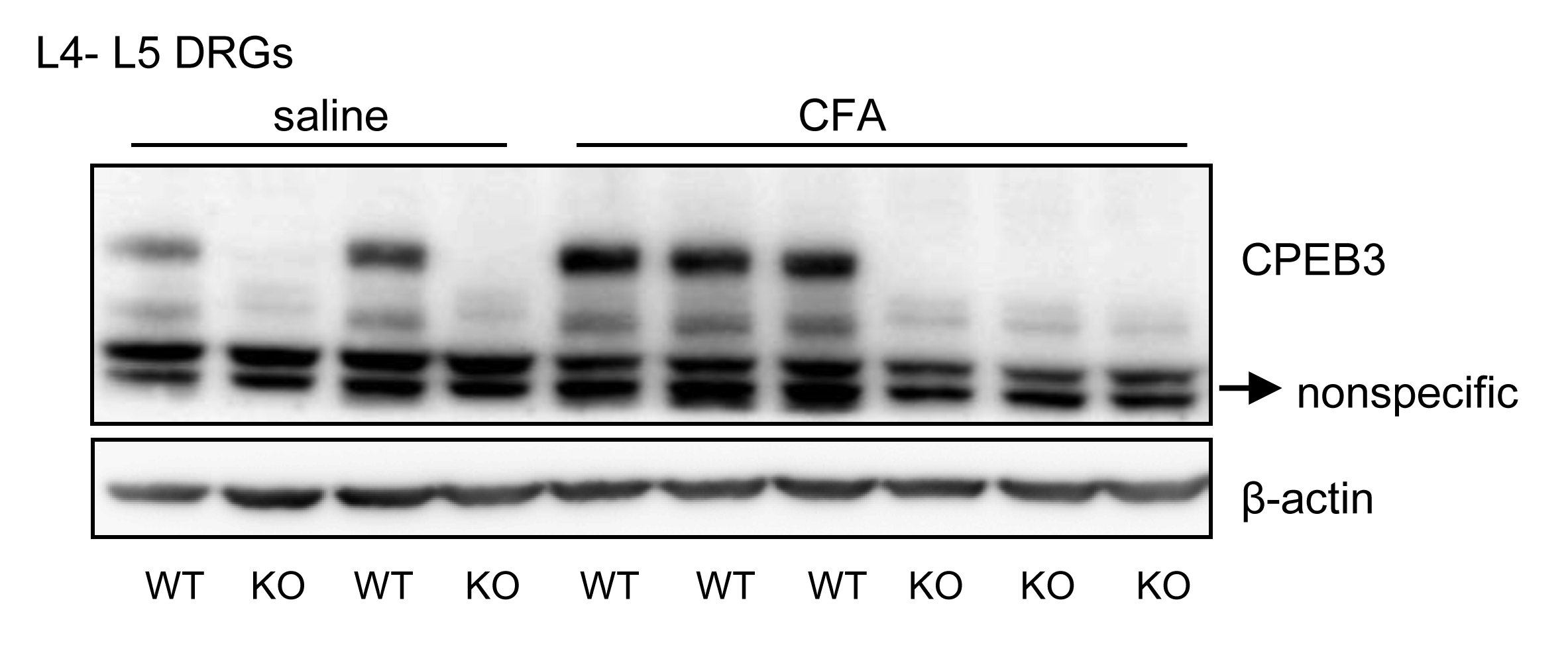

Supplement: S4 Fig — The hindpaws of WT and KO male mice were injected with saline or CFA. L4-L5 DRG were isolated a week later for immunoblotting of CPEB3 and β-actin. (TIF) [file pone.0148491.s004.tif]

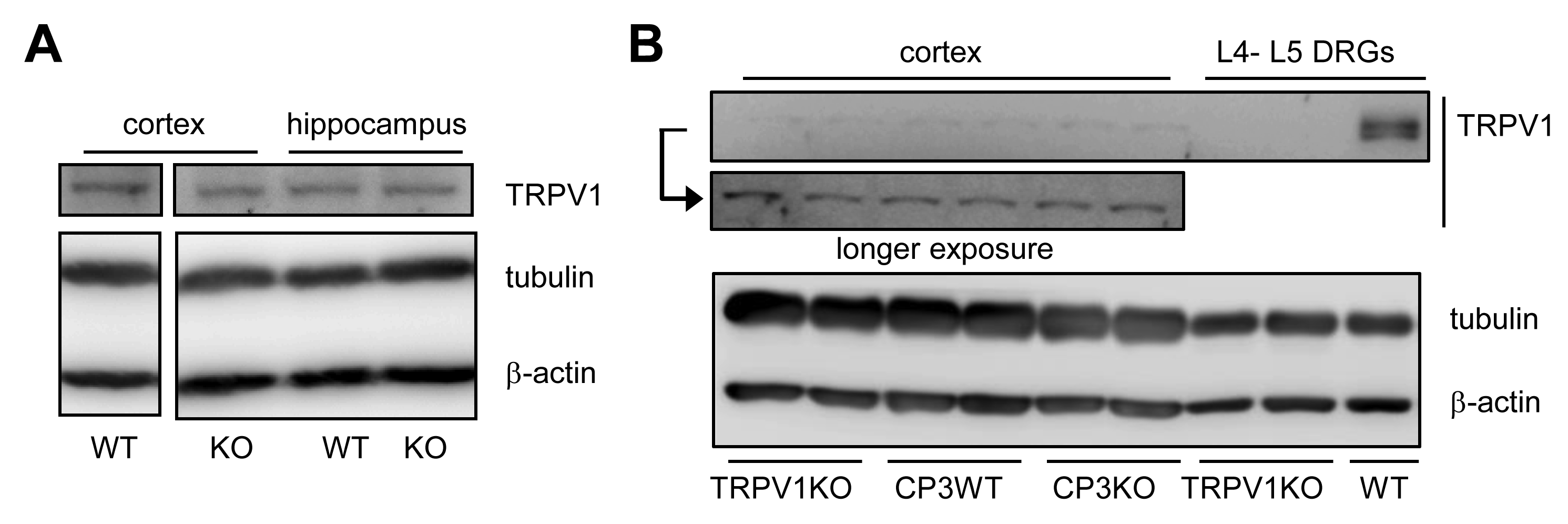

Supplement: S5 Fig — (A) Hippocampal and cortical tissues isolated from CPEB3 WT and KO male mice were used for immunoblotting with TRPV1, tubulin and β-actin antibodies. TRPV1 signals in WT and KO tissues were comparable. (B) Although TRPV1-immunoreactive signals in cortex and DRG were of the same molecular weight, the signal in the cortex was non-specific and still present in the TRPV1 KO cortex. (TIF) [file pone.0148491.s005.tif]
